# Supplementary material for: H3K9 methylation extends across natural boundaries of heterochromatin in the absence of an HP1 protein
Source: EMBO J. 2015 Oct 5;34(22):2789–803. doi: 10.15252/embj.201591320 (PMC4682641; doi:10.15252/embj.201591320)
Supplement: Supplementary file 1 — Appendix [file EMBJ-34-2789-s001.pdf]

APPENDIX

Appendix Table S1 - Strains used in this study

Strain	Genotype	Source
SPB88*	*h+ leu1-32 ura4-D18 ori1 ade6-216 imr1R(Nco1)::ura4+ swi6D::natMX*	1
SPB1779	*h90 mat3::ura4+ ura4-DS/E leu1-32 ade6-M210 swi6-linker-EGFP::hphMX*	1
SPB1780	*h90 mat3::ura4+ ura4-DS/E leu1-32 ade6-M210 nls-swi6-linker-EGFP::hphMX*	1
SPB1781	*h90 mat3::ura4+ ura4-DS/E leu1-32 ade6-M210 nls-swi6-KR25A-linker-EGFP::hphMX*	1
SPB1782	*h90 mat3::ura4+ ura4-DS/E leu1-32 ade6-M210 nls-swi6-KR25A-linker-EGFP::hphMX cnp1::mcherry::kanMX*	1
SPB1785	*h90 mat3::ura4+ ura4-DS/E leu1-32 ade6-M210 swi6-linker-EGFP::hphMX taz1::mcherry::KanMX*	1
SPB1811	*h90 mat3::ura4+ ura4-DS/E leu1-32 ade6-M210 swi6-linker-EGFP::hphMX cid14D::natMX*	1
SPB1923	*h90 mat3::ura4+ ura4-DS/E leu1-32 ade6-M210 swi6-linker-EGFP::hphMX cnp1::mcherry::kanMX*	1
SPB2001	*h90 mat3::ura4+ ura4-DS/E leu1-32 ade6-M210 nls-swi6-KR25A-linker-EGFP::hphMX taz1::mcherry::kanMx*	1
SPB2026	*h90 mat3::ura4+ ura4-DS/E leu1-32 ade6-M210 nls-swi6-linker-EGFP::hphMX cnp1::mcherry::kanMX*	1
SPB2027	*h90 mat3::ura4+ ura4-DS/E leu1-32 ade6-M210 nls-swi6-linker-EGFP::hphMX taz1::mcherry::kanMX*	1
SPB2050	*h90 mat3::ura4+ ura4-DS/E leu1-32 ade6-M210 swi6-linker-EGFP::hphMX clr4D::kanMX*	1
SPB342	*h90 mat3M(EcoRV)::gfp+::natMX ura4-DS/E leu1-32 ade6-M210*	2
SPB360	*h90 mat3M(EcoRV)::gfp+::natMX ura4-DS/E leu1-32 ade6-M210 clr4Δ::kanMX*	2
SPB939	*h90 mat3M(EcoRV)::gfp+::natMX ura4-DS/E leu1-32 ade6-M210 swi6 Δ ::ura3+*	2
SPB2361	*h90 mat3M(EcoRV)::gfp+::natMX ura4-DS/E leu1-32 ade6-M210 epe1Δ::kanMX*	1
SPB2571	*h+ leu1-32 ade6-216 ura4-D18 imr1R(NcoI)::ura4+ oriI tas3L479E-TAP-natMX*	3
SPB2594	*h90 mat3M(EcoRV)::gfp+::natMX ura4-DS/E leu1-32 ade6-M210 chp2Δ::kanMX*	1
SPB2604	*h+ leu1-32 ade6-216 ura4-D18 imr1R(NcoI)::ura4+ oriI tas3L479E-TAP-natMX swi6Δ::hphMX*	1

SPB2605	*h90 mat3M(EcoRV)::gfp+::natMX ura4-DS/E leu1-32 ade6-M210 swi6L315E*	1
SPB2610	*h90 ura4-DS/E Kint2::ura4+ swi6D::kanMX6::(swi6W104A hphMX6)*	4
SPB2611	*h90 ura4-DS/E Kint2::ura4+*	4

1 = this study, 2 = (Keller et al, 2012); *Used for plasmid expression of pmb1474
Linker amino acids in Swi6-linker-EGFP: GDGAGLIN, 3 = obtained from Danesh Moazed (Li et al, 2009), 4 = obtained from Hisao Musakata (Hayashi et al, 2009).

Appendix Table S2 - Plasmids used in this study

Name	Common name	Source
pmb85	pJR1-3xL	2
pmb1474*	pJR3xL -Swi6-linker-EGFP	1
pmb851	**pGEX - Swi6 L315E**	1

1 = this study, 2 = (Moreno et al, 2000)
* pmb1474: Swi6-linker-EGFP was cloned into pmb85 at Xho I and Spe I site. Linker amino acids: GDGAGLIN

Appendix Table S3 - Primers used in this study for ChIP-qPCR

Name	Target	Sequence	Source
mb566	*adh1+*	TCCGTTCCCCTCGAGGTT	1
mb567	*adh1+*	TCAAGGCACGATAGCAAGTGA	1
mb549	*cendg*	AAGGAATGTGCCTCGTCAAATT	1
mb550	*cendg*	TGCTTCACGGTATTTTTGAAATC	1
mb551	*cendh*	GTATTTGGATTCCATCGGTACTATGG	1
mb552	*cendh*	ACTACATCGACACAGAAAAGAAAACAA	1
mb4719	*5' of emc5+*	ATGCGTTTGCGATTCTCTGC	1
mb4720	*5' of emc5+*	GTGTGAGCGCTAACTTTTGCT	1
mb4721	*emc5+*	ACACTGCTTATTCTGCACATGA	1
mb4722	*emc5+*	TGCCGCATGTGGTAAAGACA	1
mb4509	*rad50+ (i)*	AGCCAAACTACATATATTCTCTTCATCG	1
mb4510	*rad50+ (i)*	TTGGCAGAATGTCTAGGTGTAAACTGTG	1
mb4539	*rad50+ (ii)*	ACGTACATCTTCGACTAGTTTATCCA	1
mb4540	*rad50+ (ii)*	CTATACTGGCTAACCAACTGATGACATTG	1

1 = (Keller et al, 2013)

References

Hayashi MT, Takahashi TS, Nakagawa T, Nakayama J, Masukata H (2009) The heterochromatin protein Swi6/HP1 activates replication origins at the pericentromeric region and silent mating-type locus. *Nat Cell Biol* **11:** 357-362

Keller C, Adaixo R, Stunnenberg R, Woolcock KJ, Hiller S, Buhler M (2012) HP1(Swi6) Mediates the Recognition and Destruction of Heterochromatic RNA Transcripts. *Molecular cell* **47:** 215-227

Keller C, Kulasegaran-Shylini R, Shimada Y, Hotz HR, Buhler M (2013) Noncoding RNAs prevent spreading of a repressive histone mark. *Nature structural & molecular biology* **20:** 1340

Li H, Motamedi MR, Yip CK, Wang Z, Walz T, Patel DJ, Moazed D (2009) An alpha motif at Tas3 C terminus mediates RITS cis spreading and promotes heterochromatic gene silencing. *Mol Cell* **34:** 155-167

Moreno MB, Duran A, Ribas JC (2000) A family of multifunctional thiamine-repressible expression vectors for fission yeast. *Yeast* **16:** 861-872
