# Supplementary material for: H3K9 methylation extends across natural boundaries of heterochromatin in the absence of an HP1 protein
Source: EMBO J. 2015 Oct 5;34(22):2789–803. doi: 10.15252/embj.201591320 (PMC4682641; doi:10.15252/embj.201591320)
Supplement: Supplementary file 2 — Expanded View Figures PDF [file EMBJ-34-2789-s002.pdf]

Expanded View Figures

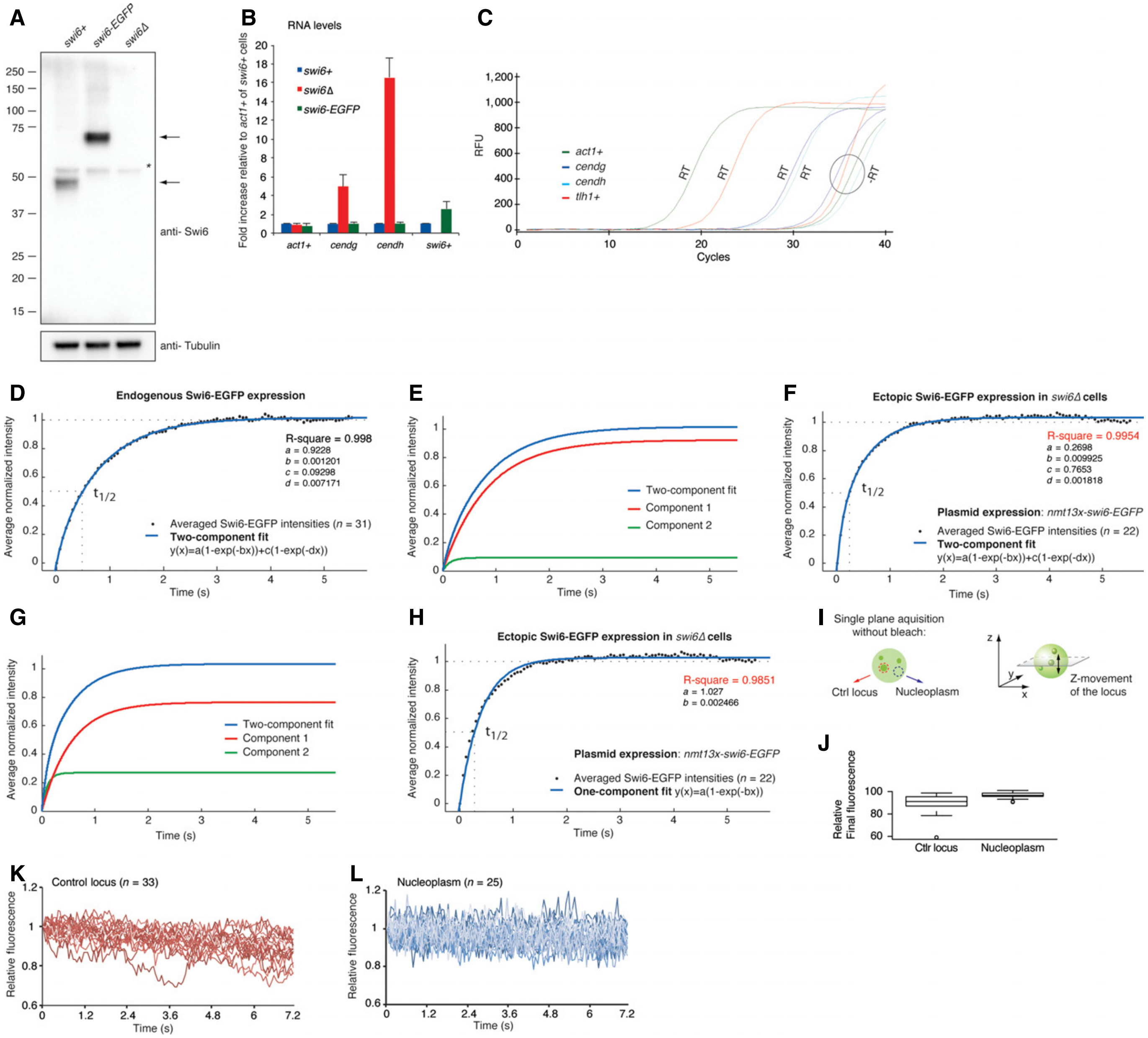

 © 2015 The Authors

Figure EV1. Dynamics of endogenously and ectopically expressed Swi6-EGFP.

A Western blot showing protein levels of endogenous Swi6 and Swi6-EGFP (denoted by arrows). The blot was probed sequentially with antibodies recognizing Swi6 and tubulin, which serves as a loading control. The asterisk indicates an unspecific band.

B Fold increase in *cendg, cendh,* and *swi6+* RNA levels in *swi6-EGFP* and *swi6Δ* cells compared with wild-type cells, relative to *act1+ mRNA*. Error bars denote s.d. from three independent experiments.

C Representative plot of real-time PCR amplification curves obtained for *act1+*, *cendg, cendh*, and *tlh1+* cDNA (RT) generated from RNA that was isolated from wild-type cells. −RT serves as a DNA contamination control (no reverse transcriptase added to the cDNA synthesis reaction). RFU, relative fluorescence units.

D Two-component fit (blue line) of normalized average FRAP intensities (black dots) obtained for endogenously tagged Swi6-EGFP at heterochromatic loci. Dashed lines indicate the final relative fluorescence intensity that is set to 1 and the fluorescence half-recovery time ($t_{1/2}$).

E Plot of individual components of the FRAP curve fit in (A). Component 1: $a(1\text{-exp}(\text{-}bx))$, Component 2: $c(1\text{-exp}(\text{-}dx))$.

F Two-component fit (blue line) of normalized average FRAP intensities (black dots) obtained for plasmid-borne ectopically expressed Swi6-EGFP at heterochromatic loci.

G Plot of individual components of the curve fit in (D). Component 1: $a(1-\exp(-bx))$, Component 2: $c(1-\exp(-dx))$.

H One-component fit (blue line) of normalized average FRAP intensities (black dots) obtained for plasmid-borne ectopically expressed Swi6-EGFP at heterochromatic loci.

I Schematics of a single-plane acquisition of a heterochromatic Swi6-EGFP locus (red, control locus) and a region in the nucleoplasm (blue). Movement of the locus out of the focal plane is depicted on the right.

J Relative final fluorescence signal after photobleaching obtained for heterochromatic and nucleoplasmic Swi6-EGFP. The box bounds the interquartile range (IQR) divided by the median, and whiskers extend to a maximum of 1.5 × IQR beyond the box.

K, L Relative fluorescence of heterochromatic Swi6-EGFP (red) (K) and nucleoplasmic Swi6-EGFP (blue) (L) acquired on a single plane in living cells over time. Fluorescence intensity at the beginning of image acquisition was set to 1.

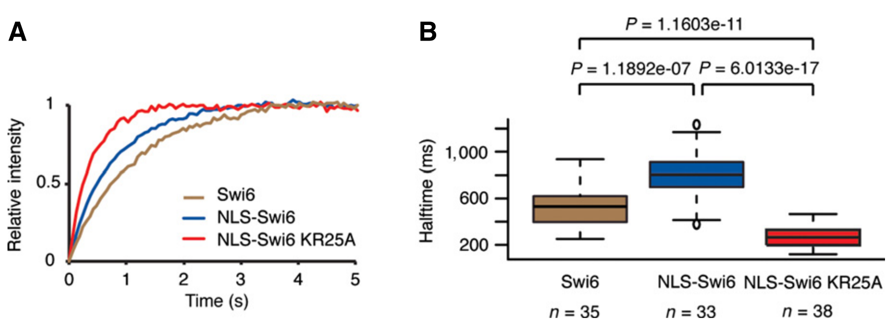

Figure EV2. Dynamics of NLS-Swi6-EGFP.

A, B Average relative intensities over time (A) and corresponding fluorescence $t_{1/2}$ values (B) of heterochromatic Swi6 obtained from FRAP experiments performed with cells expressing Swi6-EGFP (blue), NLS-Swi6-EGFP (green), or NLS-Swi6-KR25A-EGFP (red).

Data information: In (B), the box bounds the interquartile range (IQR) divided by the median, and whiskers extend to a maximum of 1.5 × IQR beyond the box.

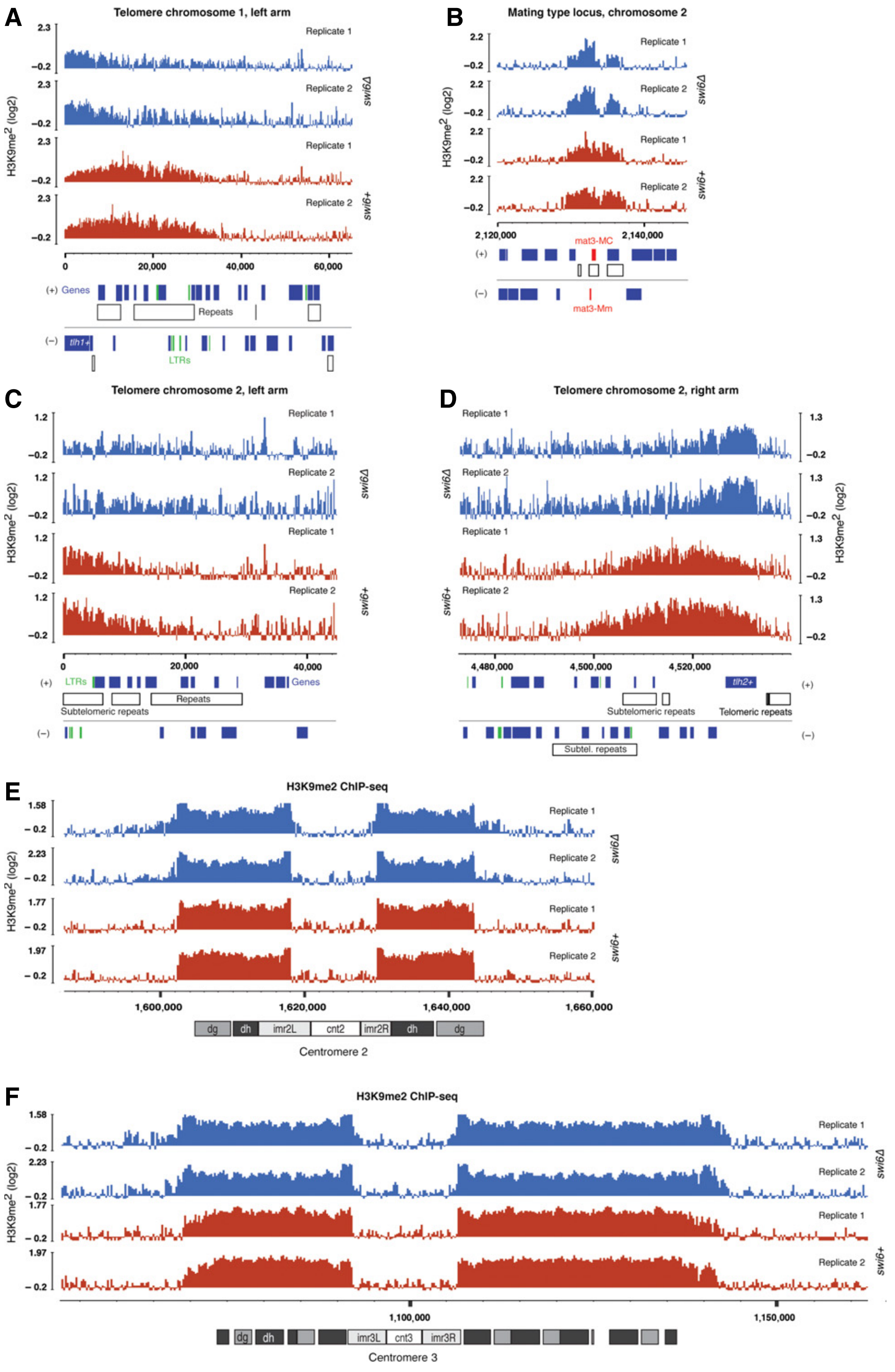

Figure EV3. H3K9 methylation in the absence of Swi6.

A, B H3K9me2 ChIP-seq enrichment profiles for *swi6+* (red) and *swi6Δ* (blue) cells at the telomere of the left arm of chromosome 1 (A) and the silent mating type locus on chromosome 2 (B).

C, D H3K9me2 ChIP-seq enrichment profiles for *swi6+* (red) and *swi6Δ* (blue) cells at the telomeres of the left (C) and right (D) arms of chromosome 2.

E, F H3K9me2 ChIP-seq enrichment profiles for *swi6+* (red) and *swi6Δ* (blue) cells at centromere 2 (E) and centromere 3 (F).

Data information: The *y*-axes represent $\log_2$ ChIP-seq enrichments in 200-bp genomically tiled windows calculated over *clr4Δ* cells. Two independent biological replicates for each genotype were performed (replicates 1 and 2). Positions of genomic elements on the plus and minus strands are indicated. Blue, protein-coding genes; white, repeats; green, long terminal repeat (LTR).

 © 2015 The Authors

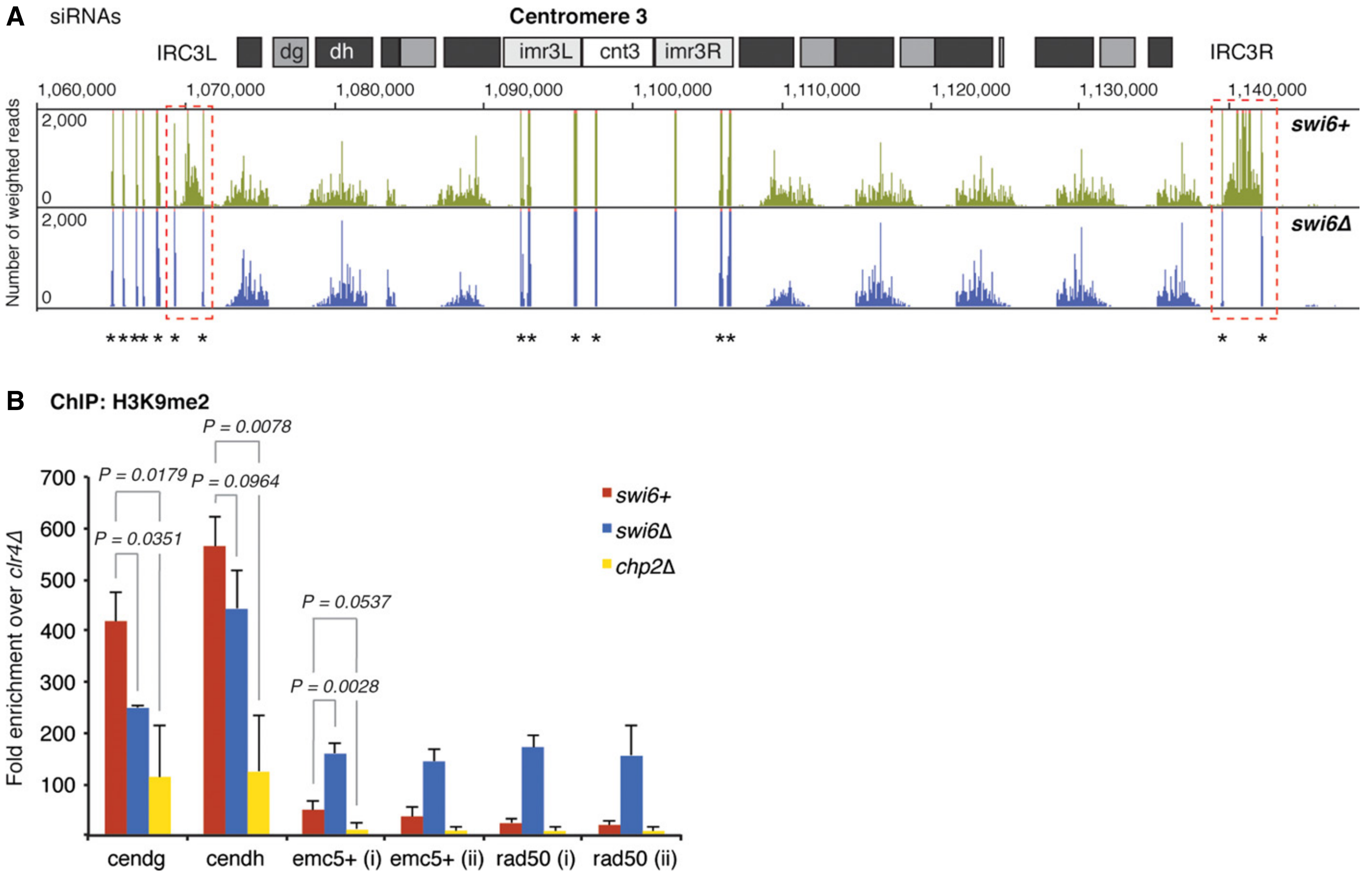

Figure EV4. siRNA levels and spreading of H3K9me at centromere 3 in the absence of Swi6.

A Small RNA reads mapping to centromere 3 in *swi6+* (green) and *swi6Δ* (blue) cells. The dashed red box points out the loss of siRNAs mapping to IRC3 elements in the absence of Swi6. Centromeric repeat elements and the central core are indicated. Counts were normalized to the library size. Asterisks denote tRNA fragments. Note that tRNA genes flank IRC3 but not IRC1 (Fig 6).

B H3K9me2 levels on heterochromatin-adjacent genes assessed by ChIP–PCR in *swi6+*, *swi6Δ*, and *chp2Δ* cells. Enrichments over *clr4Δ* were normalized to *adh1+*. Average fold enrichment with s.d. is shown for three independent experiments. *P*-values were generated by the Student's *t*-test (two-tailed distribution, two-sample, unequal variance).

© 2015 The Authors

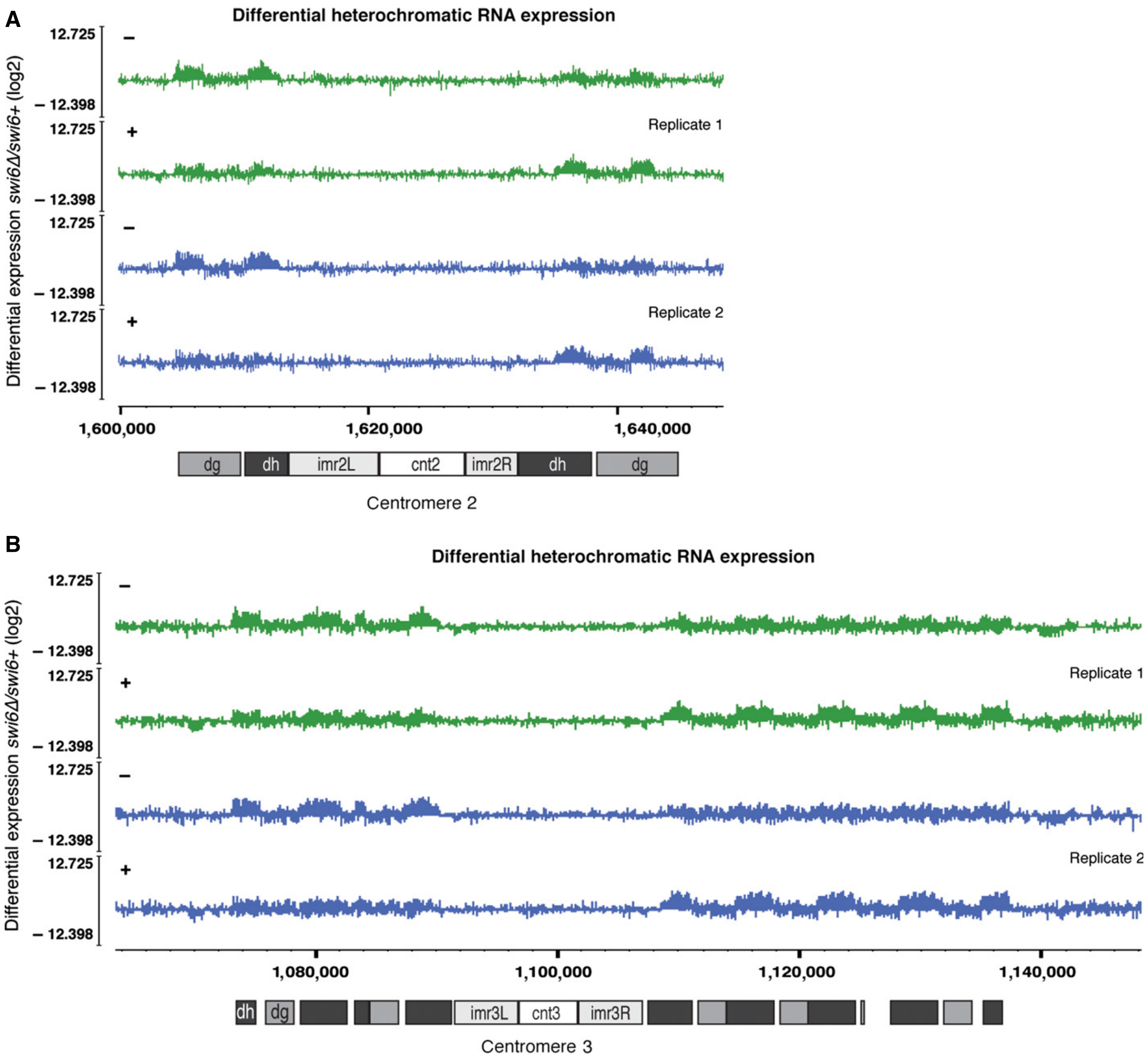

Figure EV5. Derepression of heterochromatin in the absence of Swi6.

A, B Differential expression of centromeric transcripts assessed by tiling microarray gene expression analysis. Expression data for *swi6+* and *swi6*Δ cells were taken from a previous publication (Woolcock *et al*, 2012). A, centromere 2; B, centromere 3. Different genomic elements in these regions are indicated.

 © 2015 The Authors
