# Supplementary material for: H3K9 methylation extends across natural boundaries of heterochromatin in the absence of an HP1 protein
Source: EMBO J. 2015 Oct 5;34(22):2789–803. doi: 10.15252/embj.201591320 (PMC4682641; doi:10.15252/embj.201591320)
Supplement: Supplementary file 3 — Movie EV1 [file EMBJ-34-2789-s003.zip › EMBOJ_91320_Movie_EV1_and_legend/Movie EV1 legend.rtf]

Movie EV1 – Swi6-EGFP dynamicsMovie of a FRAP experiment performed with a cell that expresses endogenouslytagged Swi6-EGFP at 0.96x speed (62.5ms/frame displayed at 16fps). The bleachoccurs in frame 6.
